# The *Leishmania* metaphylome: a comprehensive survey of *Leishmania* protein phylogenetic relationships

**DOI:** 10.1186/s12864-015-2091-2

**Published:** 2015-10-30

**Authors:** Hugo O. Valdivia, Larissa L. S. Scholte, Guilherme Oliveira, Toni Gabaldón, Daniella C. Bartholomeu

**Affiliations:** Laboratório de Imunologia e Genômica de Parasitos, Instituto de Ciências Biológicas, Universidade Federal de Minas Gerais, Av. Presidente Antonio Carlos, 6627 – Pampulha, Belo Horizonte, MG 31270-901 Brazil; Department of Parasitology, U.S. Naval Medical Research Unit No. 6, Lima, Peru; Centro de Investigaciones Tecnológicas, Biomédicas y Medioambientales, Lima, Peru; Genomics and Computational Biology Group, Centro de Pesquisas René Rachou, Belo Horizonte, Brazil; Instituto Tecnológico Vale – ITV, Belém, Brazil; Bioinformatics and Genomics Programme, Centre for Genomic Regulation (CRG), Barcelona, Spain; Universitat Pompeu Fabra (UPF), Barcelona, Spain; Institució Catalana de Recerca i Estudis Avançats (ICREA), Barcelona, Spain

**Keywords:** Phylogenomics, *Leishmania*, Homology prediction

## Abstract

**Background:**

Leishmaniasis is a neglected parasitic disease with diverse clinical manifestations and a complex epidemiology. It has been shown that its parasite-related traits vary between species and that they modulate infectivity, pathogenicity, and virulence. However, understanding of the species-specific adaptations responsible for these features and their evolutionary background is limited. To improve our knowledge regarding the parasite biology and adaptation mechanisms of different *Leishmania* species, we conducted a proteome-wide phylogenomic analysis to gain insights into *Leishmania* evolution.

**Results:**

The analysis of the reconstructed phylomes (totaling 45,918 phylogenies) allowed us to detect genes that are shared in pathogenic *Leishmania* species, such as calpain-like cysteine peptidases and 3'a2rel-related proteins, or genes that could be associated with visceral or cutaneous development. This analysis also established the phylogenetic relationship of several hypothetical proteins whose roles remain to be characterized. Our findings demonstrated that gene duplication constitutes an important evolutionary force in *Leishmania*, acting on protein families that mediate host-parasite interactions, such as amastins, GP63 metallopeptidases, cathepsin L-like proteases, and our methods permitted a deeper analysis of their phylogenetic relationships.

**Conclusions:**

Our results highlight the importance of proteome wide phylogenetic analyses to detect adaptation and evolutionary processes in different organisms and underscore the need to characterize the role of expanded and species-specific proteins in the context of *Leishmania* evolution by providing a framework for the phylogenetic relationships of *Leishmania* proteins.

Phylogenomic data are publicly available for use through PhylomeDB (http://www.phylomedb.org).

**Electronic supplementary material:**

The online version of this article (doi:10.1186/s12864-015-2091-2) contains supplementary material, which is available to authorized users.

## Background

Leishmaniasis is a group of neglected tropical diseases caused by protozoan parasites belonging to the genus *Leishmania*. The disease is present in 98 countries causing more than 1.5 million cases per year [1, 2] and posing 350 million people at risk of infection [3].

*Leishmania* belongs to the Trypanosomatidae family that is composed of obligatory parasitic organisms. Members of this family can parasitize insects as their hosts, including monoxenic organisms such as *Crithidia, Leptomonas, Herpetomonas* and *Blastocrithidia*, whereas others can also parasitize vertebrates, such as in the digenetic genera *Trypanosoma* and *Leishmania*, or plants in the genera *Phytomonas* [4]*.*

The *Leishmania* genus presents great phenotypic diversity represented by more than 30 different species, of which at least 20 are pathogenic to humans [5]. Phylogenetic analyses of the genus has further divided it into three subgenera named *Leishmania*, *Viannia* and *Sauroleishmania* [6–8].

The *Leishmania* subgenus is distributed throughout the Old and New Worlds, and it is transmitted by the bite of infected female sand flies of the genus *Phlebotomus* (Old World) and *Lutzomyia* (New World). The *Viannia* subgenus is exclusively found in the New World and is only transmitted by *Lutzomyia* sand flies [6]. In both subgenera, parasites are present as intracellular amastigotes inside phagolysosomes of phagocytes in the vertebrate host or as promastigote forms in the insect vector.

The *Sauroleishmania* subgenus that is present in the Old World is composed of non-human pathogenic *Leishmania* and it is assumed that it infects lizards through ingestion of infected *Sergentomya* sand flies [9]. Parasites of this subgenus are found as extracellular promastigotes or amastigote-like forms infecting monocyte-like cells or erythrocytes [6, 7, 10].

Leishmania parasites cause a wide spectrum of clinical manifestations that are classified into cutaneous (CL), mucosal (ML) and visceral leishmaniasis (VL). Previous studies have shown that clinical manifestation and treatment needs are associated with the infecting *Leishmania* species and the host immune response [11].

CL is primarily caused by *Leishmania (Leishmania) major, L. (Leishmania) mexicana, L. (Viannia) braziliensis* and other species of the Viannia subgenus. ML occurs in approximately 5 % of individuals with previous CL, most of who were infected with *L. (Viannia) braziliensis* [12]. VL is caused by *L. (Leishmania) infantum* and *L. (Leishmania) donovani*, which are included within the *L. donovani* complex [2].

Parasite-related factors modulate infectivity, pathogenicity, and virulence [2]. Promastigote virulence factors mediate invasion during the initial steps of an infection. For instance, lipophosphoglycan affects macrophage and dendritic cell functions and gp63 protects against complement mediated lysis and facilitates invasion [2, 13].

Candidate virulence factors in visceralizing parasites include the A2 gene family. This family has been detected in *L. (Leishmania) infantum*, *L. (Leishmania) donovani* and, as a non-expressed pseudogene, in the *L. (Leishmania) major* genome*.* All members of the A2 gene family are highly expressed during the amastigote stage, potentially allowing parasite survival at higher temperatures in visceral organs [14].

Over the last decade, *Leishmania* genome sequencing projects have resulted in the availability of a great amount of molecular data, including the genomes of *L. (Leishmania) major* Friedlin [15], *L. (Leishmania) infantum* JPCM5, *L. (Viannia) braziliensis* M2904 [16], *L. (Leishmania) amazonensis* M2269 [7] and several others draft assemblies that are available to the scientific community [17].

Comparative genomic studies have reported high synteny across *Leishmania* species despite a breach of 36–46 million years divergence between New World and Old World species [18]. Only 200 genes with differential distributions across *L. (Leishmania) major, L. (Leishmania) infantum,* and *L. (Viannia) braziliensis* have been described based on sequence similarity [16].

The identification of homologous genes is a critical step to understand the evolutionary history of an organism. Homologs can be divided into two types: orthologs, which originated through a speciation event from a common ancestor and paralogs, which resulted from a duplication event [19–21]. This classification is critical to understanding the diversification processes because duplication events are often related to a posterior functional divergence [22, 23].

Accurate predictions of homology relationships can be used to infer gene functionality [22], reconstruct species phylogenies, and characterize genomes based on their encoded genes [19]. For these purposes, different methods have been proposed. Most of them rely on sequence similarity between genes where function and homology are assessed from the most similar sequences [22]. These methods are fast; however, they have drawbacks because sequence similarity does not always have a direct relationship to functionality [22].

Phylogenomics, which analyzes genomic information in the context of its evolution, is a promising method for inferring homology relationships [24, 25]. This method establishes homology from an evolutionary perspective rather than relying only on sequence similarity [22]. It has also been previously used to reveal the origin and evolution of phenotypic characteristics and further our knowledge of metabolism, pathogenicity, and adaptation of an organism to its surroundings [24, 26–28].

In the current study, we employed a phylogenomics-based approach to analyze the phylomes of six *Leishmania* species to study their evolution and provide a comprehensive survey of the phylogenetic history of all proteins in *Leishmania*.

## Methods

### Sequence data

Predicted proteomes from six *Leishmania* species Predicted proteomes from six *Leishmania* species (*L. (Viannia) braziliensis*, *L. (Leishmania) mexicana, L. (Leishmania) major*, *L. (Leishmania) infantum, L. (Leishmania) donovani*, *Leishmania (Sauroleishmania) tarentolae*) and *Trypanosoma brucei* were downloaded from the TritrypDB V5 [17] (Table 1). Prior to the analysis, proteome data were filtered with a customized Perl script to select proteins starting with methionine, lacking internal stop codons, represented by the 20 IUPAC amino acid codes, and longer than 100 amino acids.

**Table 1:** Proteomes selected for the construction of *Leishmania* phylomes

Species	NCBI ID	Total proteins	Valid proteins		Trees generated	Proteome coverage (%)
			#	%		
*L. (Viannia) braziliensis*	420245	8357	7942	95.0	7712	97.1
*L. (Leishmania) donovani*	981087	8033	7736	96.3	7550	97.6
*L. (Leishmania) infantum*	435258	8238	7974	96.8	7808	97.9
*L. (Leishmania) major*	347515	8400	8170	97.3	7849	96.1
*L. (Leishmania) mexicana*	929439	8250	7953	96.4	7796	98.0
*L. (Sauroleishmania) tarentolae*	5689	8452	7465	88.3	7203	96.5

### Phylome reconstruction

**Fig. 1:**
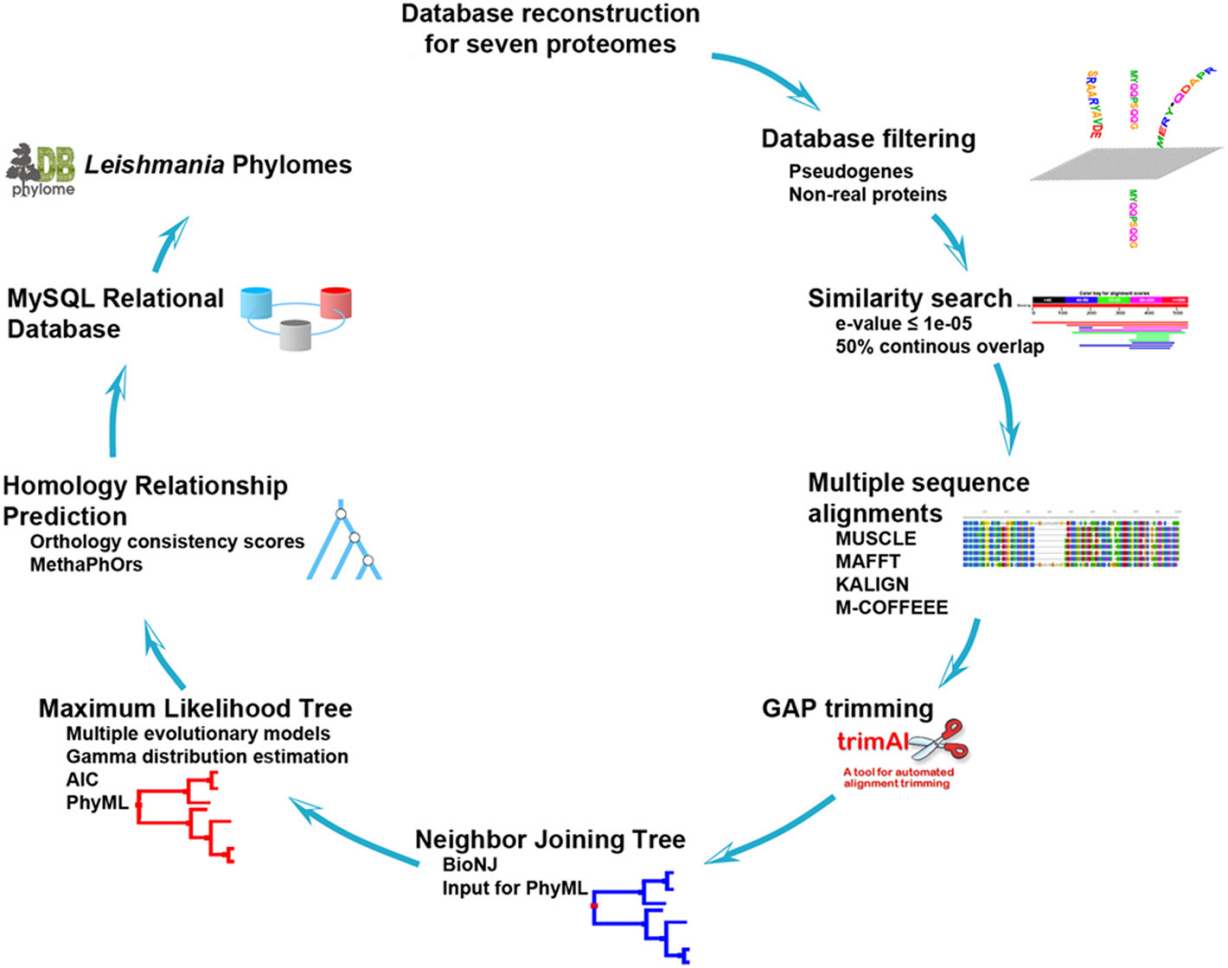
Phylogenomics pipeline. Each protein was treated as a seed and compared against all proteins encoded in the database. Groups of similar proteins were aligned and trimmed to remove gap-enriched regions. The trimmed alignment was used to build a NJ tree, which was then employed to create a maximum likelihood tree using the two best evolutionary models selected by AIC. Lineage specific duplications and homology relationships were determined and a relational database was created to store and analyze phylomic data

Phylome reconstruction for all species was done following an automated pipeline that was previously described [29] (Fig. 1). Briefly, a local database was created comprising all proteomic data. For each protein sequence (seed), a Smith-Waterman search [30] was performed against the aforementioned database to retrieve highly similar proteins with a continuous alignment length of more than 50 % of the query sequence and e-value ≤ 1e-05.

Sets of similar protein sequences were aligned using MUSCLE v3.8 [31], MAFFT v6.712b [32] and KALIGN v2.04 [33]. Alignments were performed in the forward and reverse directions and combined using M-COFFEE [34]. Gaps were removed from the final alignment using trimAl v1.3 [35] with a consistency and gap score cutoffs of 0.1667 and 0.1, respectively.

Neighbor-joining trees were constructed for each trimmed alignment as implemented in BioNJ [36], and *T. brucei* protein sequences were used as the out-group. The resulting NJ tree was used as input for PhyML v3.0 [37] to create a maximum likelihood tree, allowing branch length optimization using different evolutionary models (JTT, LG, WAG, Blosum62, MtREV, VT and Dayhoff).

The two evolutionary models that better modeled the data were determined according to the Akaike Information Criterion (AIC) [38]. Maximum likelihood trees were derived using the two selected models. In all cases, we used a discrete gamma-distribution model with four rate categories plus invariant positions; the gamma parameter and fraction of invariant positions were estimated from the data. Tree support values were calculated with an approximate likelihood ratio test (aLTR) in PhyML [37].

All phylome-related data, including trees and alignments, can be downloaded and browsed through PhylomeDB [39] (www.phylomedb.org).

### Detection of homology relationships

To identify orthologs and paralogs, we used a species-overlap algorithm [27] as implemented in the environment for tree exploration (ETE) v2 [40]. Shortly, this algorithm starts at each seed protein used for generating the tree and traverses it until reaching the root. Each internal node was labeled as a duplication or speciation event, depending on whether their daughter partitions showed genes from the same or different species.

Orthology and paralogy relationships derived from the analyses of each phylome were combined into a single prediction using the MetaPhOrs algorithm [41] with a cutoff consistency score of 0.5, meaning that orthology relationship between two genes is called if the majority of examined trees containing these two sequences are consistent with this prediction.

### Detection of species-specific expansions

We analyzed the *Leishmania* methaphylome using ETE to identify families that were specifically expanded in each species since their diversification. For this purpose, we considered those duplications detected by the species overlap algorithm that only comprised paralogs as species-specific expansions. An in-house Perl script was subsequently used to filter out redundant paralogous and orthologous proteins and load them into a MySQL relational database.

Gene Ontology codes that were significantly overrepresented in expanded families were detected using the hypergeometric distribution analysis in BiNGO [42] with Benjamini and Hochberg false discovery rate correction (corrected *p* value <0.05).

## Results and discussion

### Phylome reconstruction

The *Leishmania* metaphylome was derived from comparative analyses of all proteins encoded by six *Leishmania* species and *Trypanosoma brucei*, which was included as the out-group. The selected set of species includes causal agents of CL (*L. (Viannia) braziliensis*, *L. (Leishmania) mexicana* and *L. (Leishmania) major*), ML (*L. (Viannia) braziliensis*), VL (*L. (Leishmania) infantum* and *L. (Leishmania) donovani*) and a non-human pathogenic *Leishmania* (*L. (Sauroleishmania) tarentolae*).

From an initial set of 49,730 *Leishmania* proteins, 47,240 (94.9 %) were analyzed after filtering for valid sequences resulting in 45,918 phylogenetic trees summarizing the evolutionary relationships of 46,667 proteins (98.8 % of all valid proteins). This coverage is greater than the ones obtained for other phylomes such as the *Schistosoma mansoni* (70 %) [24] or the pea aphid *Acyrthosiphon pisum* (67 %) [26], thereby underscoring the high quality and sequence conservation of the datasets.

The absence of trees for the remaining 573 proteins could be due to high divergence between these proteins and their homologs in the dataset. Alternatively, this set of remaining proteins may include species-specific proteins that did not present homologs due to their uniqueness (Additional file 1: Table S1). Finally, another possibility is the presence of errors in the gene models as has been previously suggested [24].

### Species-specific expansions

It has been shown that gene duplication plays an important role in evolution that results in increased expression or novel functionalization and/or sub-functionalization [43, 44]. Duplicated or diversified paralogs may be kept in the genome if they provide a selective advantage [27]. Therefore, inspecting the functions of expanded families may provide evidence of these processes in the evolution of *Leishmania*.

The *Leishmania* metaphylome provides an overview of protein evolutionary relationships that can be explored to reveal events related to *Leishmania* diversification and adaptation. Using the species-overlap algorithm [40], we analyzed species-specific protein expansions in all *Leishmania* proteomes and reported the most expanded proteins for each species (Table 2, Additional file 1: Table S2).

**Table 2:** Top *Leishmania* species-specific protein expansions using the species-overlap algorithm

Species	Seed	Seed annotation	Expansions
*L. (Viannia) braziliensis*	LbrM.34.0020	TATE DNA Transposon	30
*L. (Viannia) braziliensis*	LbrM.08.1140	amastin-like protein	28
*L. (Viannia) braziliensis*	LbrM.10.0520	GP63, leishmanolysin,metallo-peptidase, Clan MA(M), Family M8	25
*L. (Leishmania) major*	LmjF.12.0755	surface antigen protein 2, putative	20
*L. (Leishmania) mexicana*	LmxM.08.0750	amastin-like protein, putative	19
*L. (Sauroleishmania) tarentolae*	LtaPcontig05711-1	Hypothetical protein, unknown function	14
*L. (Sauroleishmania) tarentolae*	LtaP10.0670	Major surface protease gp63, putative;GP63, leishmanolysin	12
*L. (Leishmania) major*	LmjF.34.1720	amastin-like surface protein, putative	12
*L. (Leishmania) major*	LmjF.12.0950	hypothetical protein, conserved	11
*L. (Viannia) braziliensis*	LbrM.30.0450	histone H4	10
*L. (Leishmania) mexicana*	LmxM.08.1080	cathepsin L-like protease, putative	8
*L. (Viannia) braziliensis*	LbrM.19.1530	glycerol uptake protein, putative	7
*L. (Viannia) braziliensis*	LbrM.02.0550	Retrotransposable element SLACS	7
*L. (Leishmania) mexicana*	LmxM.12.0870partial	surface antigen protein 2, putative	7
*L. (Leishmania) major*	LmjF.09.0156	ATG8/AUT7/APG8/PAZ2, putative (ATG8C.4)	7
*L. (Leishmania) major*	LmjF.08.1030	cathepsin L-like protease	7
*L. (Leishmania) donovani*	LdBPK_100380.1	folate/biopterin transporter, putative	5
*L. (Leishmania) infantum*	LinJ.10.0520	GP63, leishmanolysin,metallo-peptidase, Clan MA(M), Family M8 (GP63-3)	5
*L. (Leishmania) infantum*	LinJ.36.0010	phosphoglycan beta 1,3 galactosyltransferase 4 (SCG4)	5

**Fig. 2:**
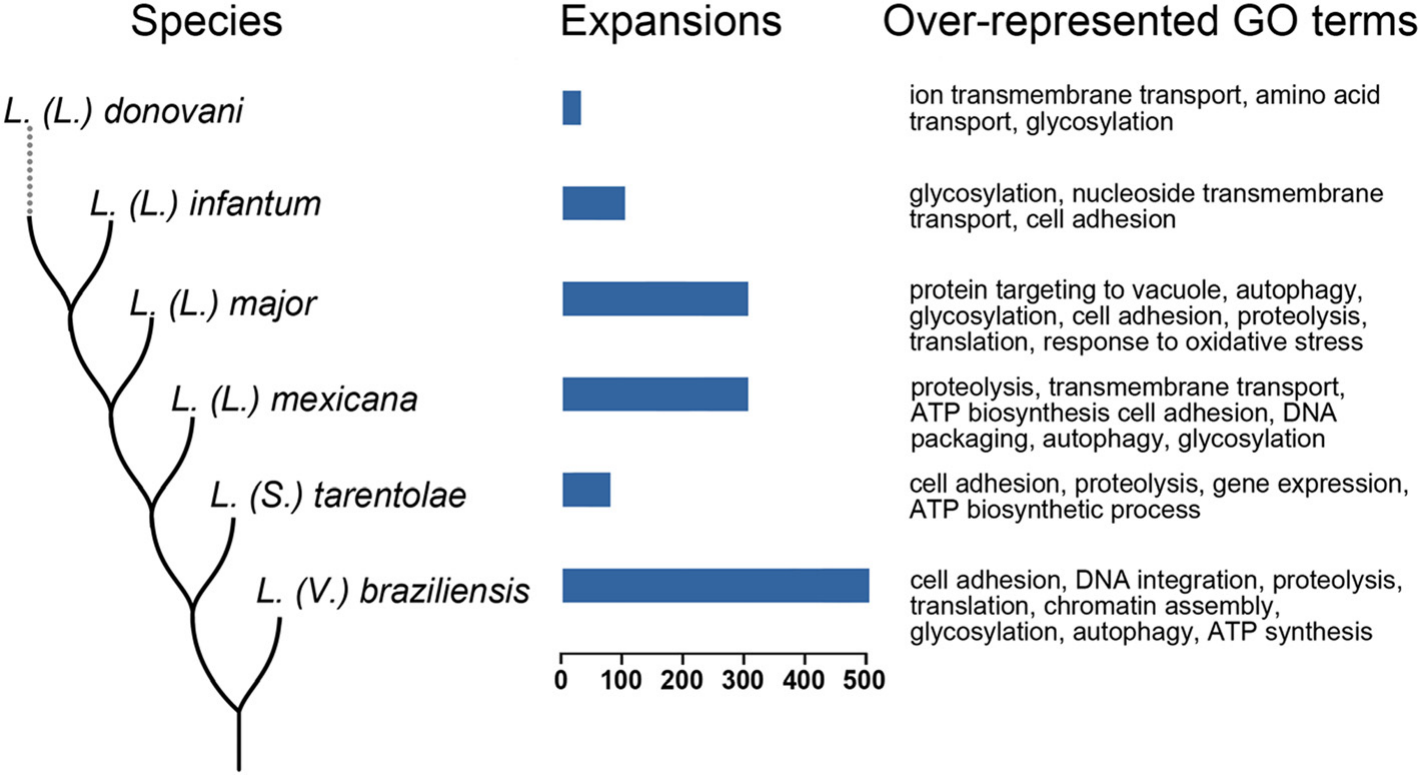
Estimates of expansions in *Leishmania. Horizontal bars* indicate the number of genes in expanded families per species. On the *right* we show significantly over-represented GO terms (corrected *p* value <0.05) compared to the rest of the genome in the set of expanded gene families

Our results show that species-specific expansions vary greatly between species with *L. (Viannia) braziliensis* and *L. (Leishmania) donovani* accumulating the highest and lowest number of expansions, respectively (Fig. 2). Expanded proteins include well characterized families such as amastins, metalloproteinases, cysteine proteases and surface antigen proteins (Additional file 1: Table S2). These families are important virulence factors in *Leishmania* and reveal an evolutionary trend towards parasitism.

Over-represented Gene Ontology terms in expanded families also show species-specific adaptations (Fig. 2). However, common over-represented terms such as “glycosylation,” “proteolysis,” “cell adhesion” and “autophagy” are consistent with adaptation towards a parasitic lifestyle.

Glycosylation appears as an important mechanism of protein modification and may play a role in protein maturation and protein function in *Leishmania* [45]. Promastigote and amastigote stages express different types of proteophosphoglycans (PPGs) on their surfaces, and changes in the glycosylation of these proteins have resulted in striking reductions in promastigote and amastigote virulence in *L. (Leishmania) major* [46].

Proteolysis is a key component of pathogenesis in *Leishmania*, acting on several host intracellular proteins such as cytoskeleton regulators, transcription factors or protein phosphatases [47, 48]. It has also been suggested that the direction of proteolytic activities towards degradative enzymes in phagolysosomes and major histocompatibility complex molecules may promote parasite survival by impairing host response and proper antigen presentation [49].

Autophagy has been shown to play an important function during *Leishmania* differentiation from procyclic to metacyclic promastigotes and into amastigotes with an increase in autophagosomes and protein degradation levels [50]. Additionally, degradation of glycosomes allows organelle renewal and enables the parasites to rapidly adapt to the new conditions within their various hosts [51].

Among the most expanded proteins in *L. (Viannia) braziliensis*, we detected the presence of TATE DNA transposons (Telomere-Associated Transposable Element) and SLACS (Spliced Leader Associated Conserved Sequence). SLACS are specific retroposons that are located between tandem arrays of spliced leader RNA genes while TATE transposons tend to be located at telomeres. These transposable elements are the source of most siRNA in *L. (Viannia) braziliensis* [52] that are generated by the RNAi machinery, which appears to be specific to the *Viannia* subgenus to downregulate the expression of mobile elements that can affect genome integrity [52].

**Fig. 3:**
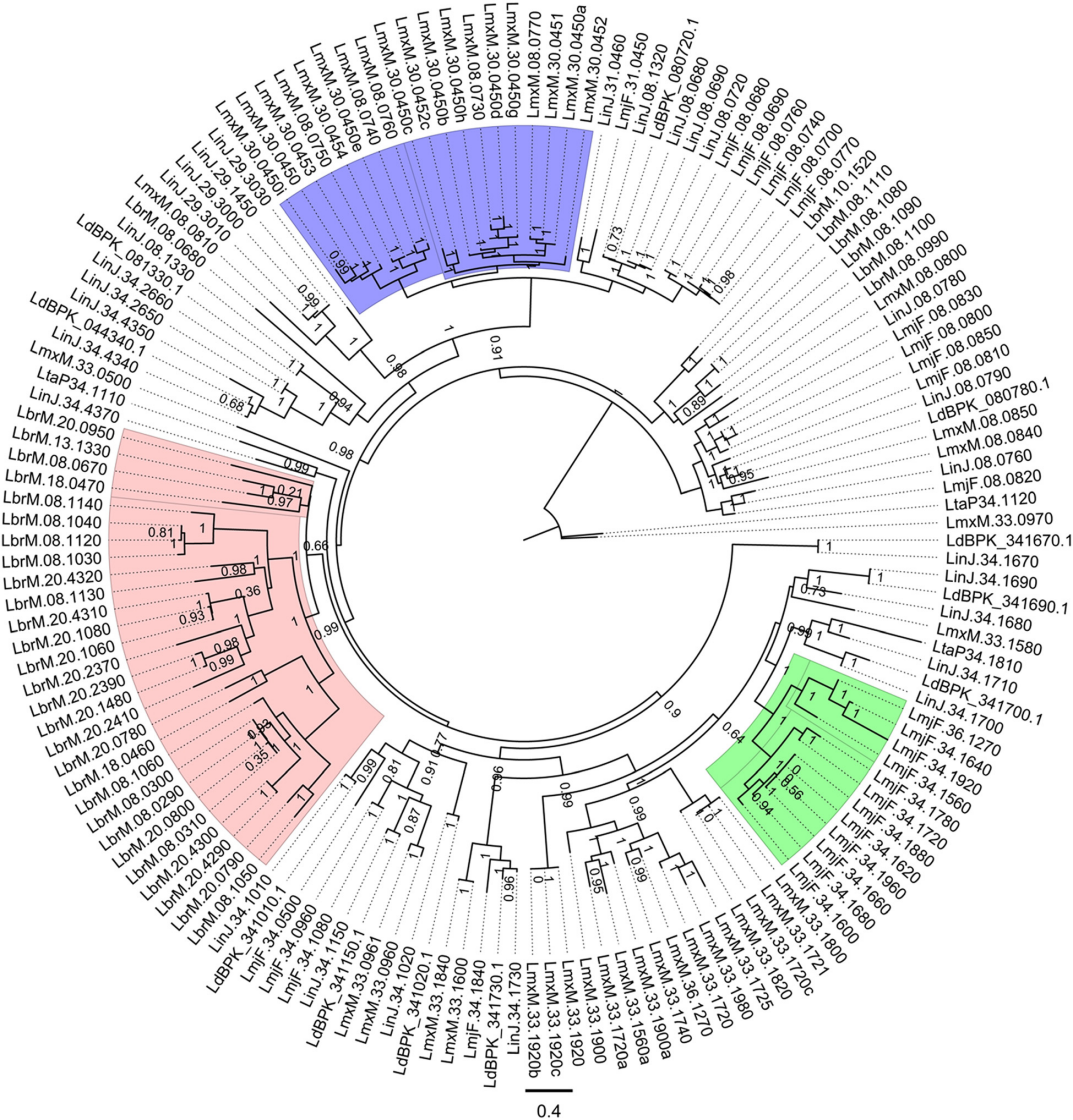
Amastin phylogenetic tree. Phylogenetic relationships of 150 Amastin protein members using *L. (Viannia) braziliensis* LbrM.08.1140 as seed protein with JTT as the best-fit model. Numbers indicate support values computed by the approximate likelihood ratio test (aLTR). Colored regions show species-specific expansions as follows: *Rose*: *L. (Viannia) braziliensis*; *Green*: *L. (Leishmania) major*; *Blue*: *L. (Leishmania) mexicana*. Gene codes indicate the following species: LinJ: *L. (Leishmania) infantum*; LmxM: *L. (Leishmania) mexicana*; LmjF: *L. (Leishmania) major*; LdBPK: *L. (Leishmania) donovani;* LbrM: *L. (Viannia) braziliensis*; Lta: *L. (Sauroleishmania) tarentolae*

Another expanded protein family in *L. (Viannia) braziliensis* is amastin. This family of surface glycoproteins is highly expressed in amastigotes and, while their exact function is not known, they appear to mediate host-parasite interactions, allowing parasite infection and survival [53]. It has been previously shown that amastins are expanded in all *Leishmania* species compared to *Trypanosoma*, suggesting a functional adaptation [53]. The corresponding amastin phylogeny of our analysis comprises only proteins that originated after *Leishmania* diversification [53] and revealed that *L. (Viannia) braziliensis, L. (Leishmania) mexicana*, and *L. (Leishmania) major* have greatly expanded their delta-amastin repertoire compared to the visceral species included in the phylogenomic analyses (Fig. 3). (For an extensive evolutionary analysis of amastins in Trypanosomatids see Jackson [53]).

We have also detected expansions in the GP63 protein family in *L. (Viannia) braziliensis* and *L. (Sauroleishmania) tarentolae* (Table 2)*.* These metallopeptidases participate in parasite interactions with both invertebrate and vertebrate hosts, ensuring parasite invasion and survival [54–57].

**Fig. 4:**
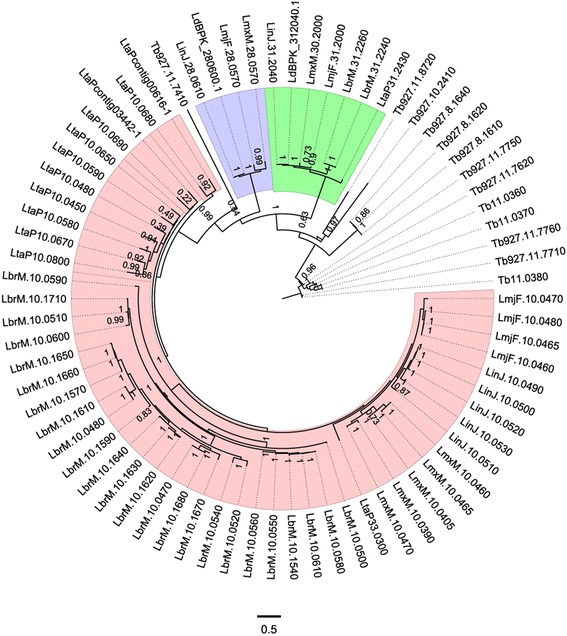
GP63 phylogenetic tree. Phylogenetic tree for GP63 using *L. (Viannia) braziliensis* seed protein LbrM.31.2240 and WAG as the best-fit model. Numbers indicate support values computed by the approximate likelihood ratio test (aLTR). Colored regions denote GP63 distribution as follows: *Green*: Chromosome 31 GP63; *Blue*: Chromosome 28 GP63; *Rose*: Chromosome 10 GP63. Gene codes indicate the following species: LinJ: *L. (Leishmania) infantum*; LmxM: *L. (Leishmania) mexicana*; LmjF: *L. (Leishmania) major*; LdBPK: *L. (Leishmania) donovani;* LbrM: *L. (Viannia) braziliensis*; Lta: *L. (Sauroleishmania) tarentolae*; Tb: *T. brucei*

To perform a deeper analysis of the GP63 phylogenomic data, we conducted a case analysis using the tree that retrieved the largest number of homologs across all species (seed “LbrM.31.2240”) (Fig. 4). This tree recovered 63 GP63 proteins from an initial set of 74 annotated GP63 plus 11 additional proteins that were annotated as hypothetical proteins in the input dataset. Proteins annotated as GP63 that were not present in this tree are shorter in length, lack a peptidase domain, or have an incorrect annotation in the proteome dataset.

GP63 genes in the *Leishmania* subgenus range from two genes in *L. (Leishmania) donovani* to seven in *L. (Leishmania) infantum* and GP63. On the contrary, the GP63 repertoire has greatly expanded in *L. (Viannia) braziliensis* and *L. (Sauroleishmania) tarentolae* reaching up to 26 and 13 genes, respectively (Fig. 4).

**Fig. 5:**
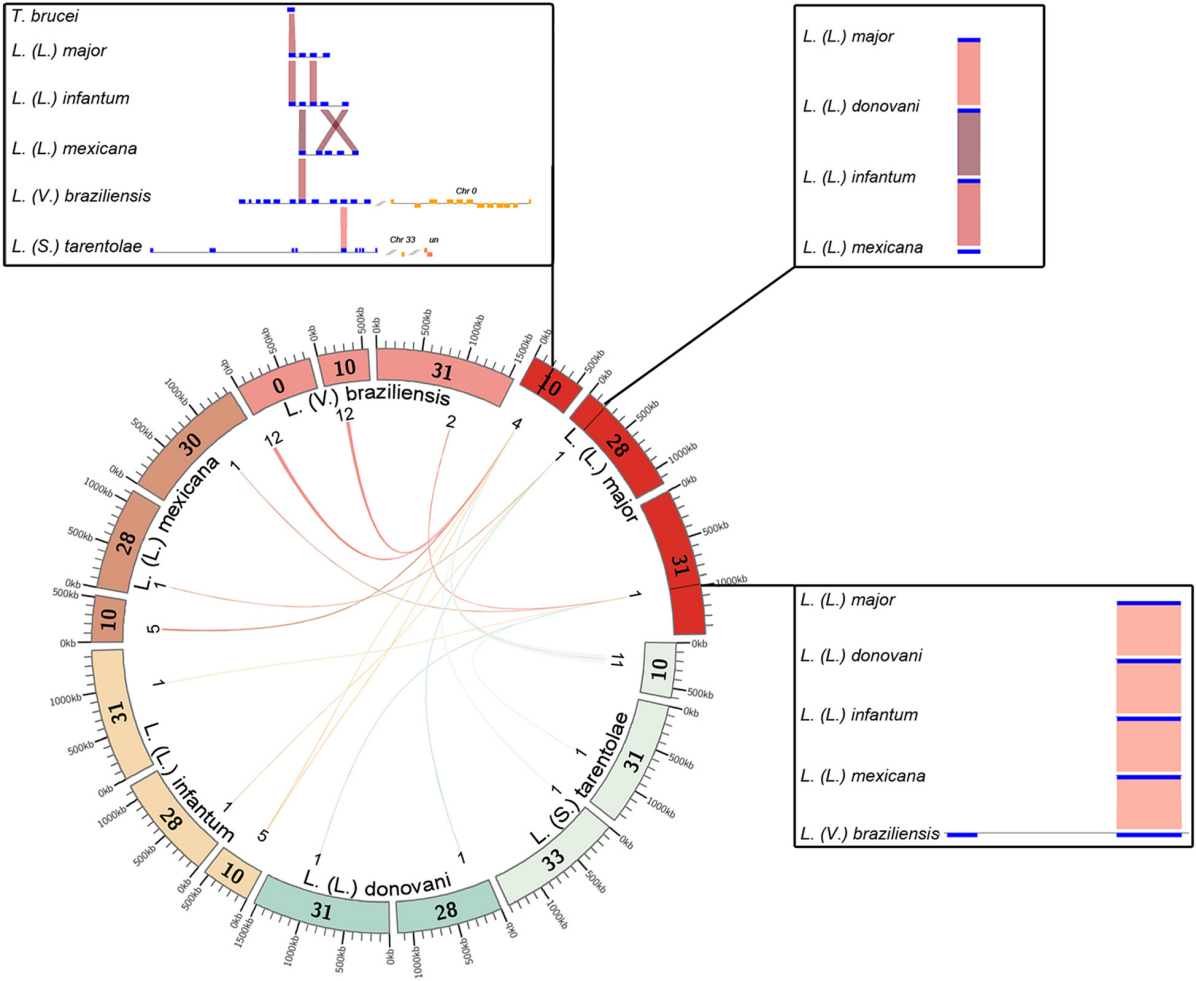
GP63 chromosome distribution. Location of GP63 genes in *Leishmania* genomes. Boxes represent each GP63 gene per species for each chromosome, while inner links show best reciprocal blast-best hit, and numbers indicate GP63 genes on the respective chromosome; Chr 0: Chromosome 0; un: undetermined. *Inner lines* indicate blast results against the *L. (Leishmania) major* GP63 genes and show the position of each gene within its respective chromosome

Our analysis shows that the GP63 family appears to have suffered expansion events at different times during Trypanosomatids’ evolution and can be divided in three distinct subfamilies located on chromosomes 31, 28, and 10 (Fig. 5). GP63 of chromosome 31 consists of a single GP63 gene present in all *Leishmania* species except *L. (Viannia) braziliensis*, where it is composed of two distinct isoforms that are located in an array (Figs. 4 and 5).

GP63 of chromosome 28 is present only in the *Leishmania* subgenus and is represented by one gene in *L. (Leishmania) major, L. (Leishmania) mexicana*, *L. (Leishmania) donovani* and *L. (Leishmania) infantum*, sharing more than 93 % similarity at the protein level.

GP63 of chromosome 10 constitutes a set of gene arrays in all *Leishmania* species except *L. (Leishmania) donovani,* where it is completely absent. The phylogeny shows that this subfamily branches with *T. brucei* GP63, supporting a common origin with subsequent gains and losses in *Leishmania* (Fig. 4). Among chromosome 10 GP63s, *L. (Sauroleishmania) tarentolae* and *L. (Viannia) braziliensis* stand out as the species with the highest number of expansions.

Alignment data for the Chr 10 subfamily revealed that *L. (Sauroleishmania) tarentolae* Chr 10 GP63 proteins are shorter than those of *L. (Viannia) braziliensis* (291 versus 560 amino acids), lack predicted extracellular regions, and have a shorter peptidase domain. These characteristics may affect parasite host interaction and limit GP63 protease activity in *L. (Sauroleishmania) tarentolae*, as has been previously suggested [7]. Another possibility could be assembly completeness of the *L. (Sauroleishmania) tarentolae* genome, which may result in partial GP63 sequences [7].

Given that the long arrays in *L. (Viannia) braziliensis* are absent from the other *Leishmania* species, it is highly possible that this expansion occurred after the origin of the *Viannia* subgenus. Interestingly, it has been previously shown that GP63 is also present in high copy number in *L. (Viannia) peruviana* and *L. (Viannia) guyanensis* [58, 59].

This information suggests that large GP63 expansions in chromosome 10 are characteristic of the *Viannia* subgenus and could respond to an adaptation mechanism to the wider range of reservoirs and vectors that the species of this subgenus infect. In the case of *L. (Sauroleishmania) tarentolae*, GP63 expansions could be related to interactions with a different genus that serves as vector (*Sergentomya*) and the lizard host*.*

**Fig. 6:**
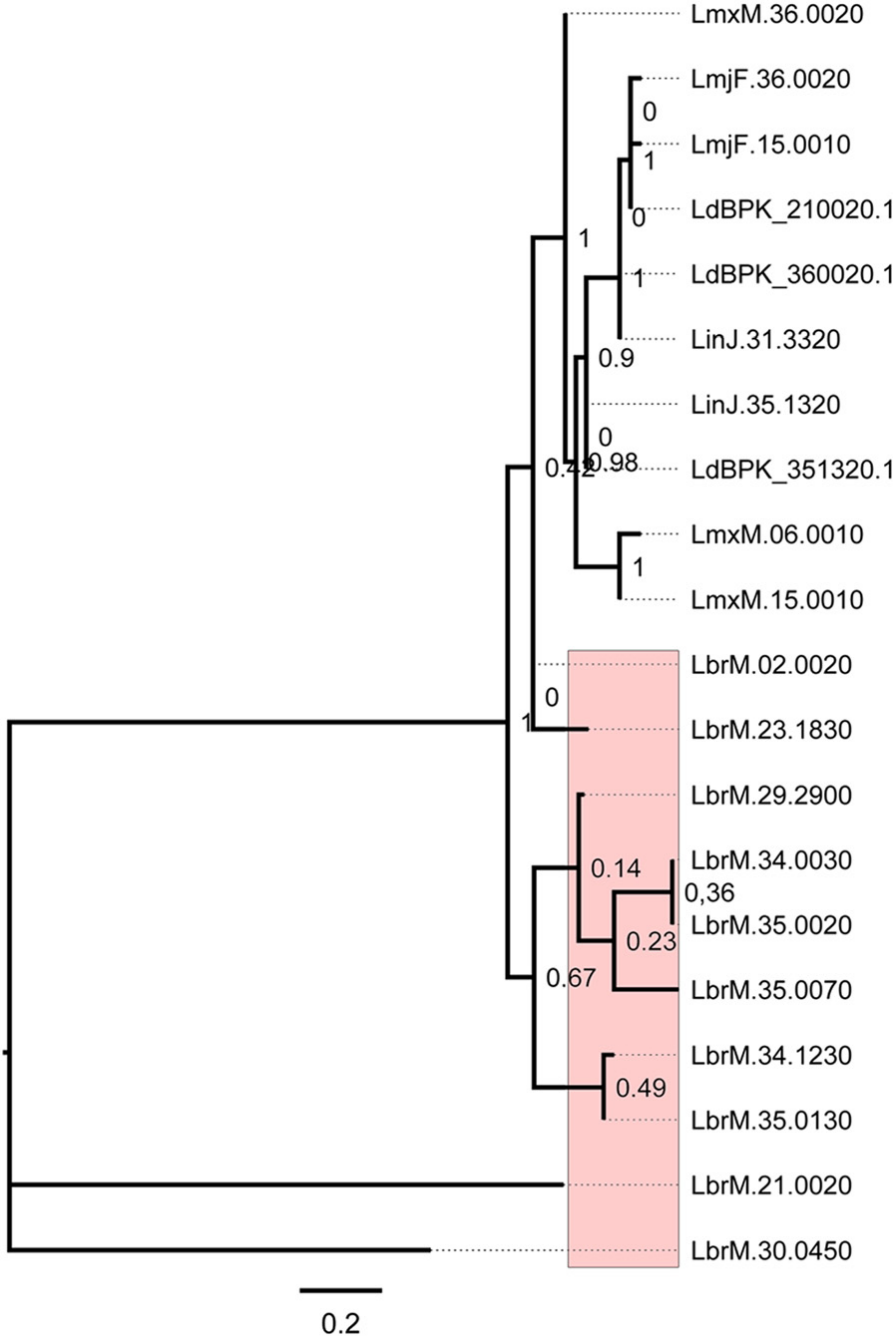
Histone 4 phylogenetic relationship. Phylogenetic relationships for Histone 4 using *L. (Viannia) braziliensis* seed protein LbrM.30.0450 and JTT as the best-fit model. Numbers indicate support values computed by the approximate likelihood ratio test (aLTR). Due to their high sequence similarity, there is a large inconsistency in most nodes as reflected in the tree. Rose-colored regions indicate *L. (Viannia) braziliensis* expansions. Gene codes indicate the following species: LinJ: *L. (Leishmania) infantum*; LmxM: *L. (Leishmania) mexicana*; LmjF: *L. (Leishmania) major*; LdBPK: *L. (Leishmania) donovani;* LbrM: *L. (Viannia) braziliensis*

Histone 4 has also been shown to be differentially expanded in *L. (Viannia) braziliensis* with 10 genes. In the *Leishmania* subgenus, Histone 4 is reduced to three or less genes and is completely absent in *Sauroleishmania* (Fig. 6). However, the lack of Histone 4 in *Sauroleishmania* could likely result from the limitations in the current genome assembly of this species.

H4 expansions in *L. (Viannia) braziliensis* are not restricted to a single chromosome, suggesting derivation of novel loci through transposition. Sequence alignment of these expansions showed a conserved core with more than 80 % sequence similarity among all sequences and the presence of variable regions at the N and C terminal ends.

Post-translational modification analysis in histones of Trypanosomatids has revealed that H4 and H3 are heavily acetylated and methylated on the N-terminal tails in *Trypanosoma*, and these modifications change during parasite development [60]. Whether expansions and diversification in histone 4 of *L. (Viannia) braziliensis* have a role in transcriptional regulation in *Leishmania* remains to be investigated.

**Fig. 7:**
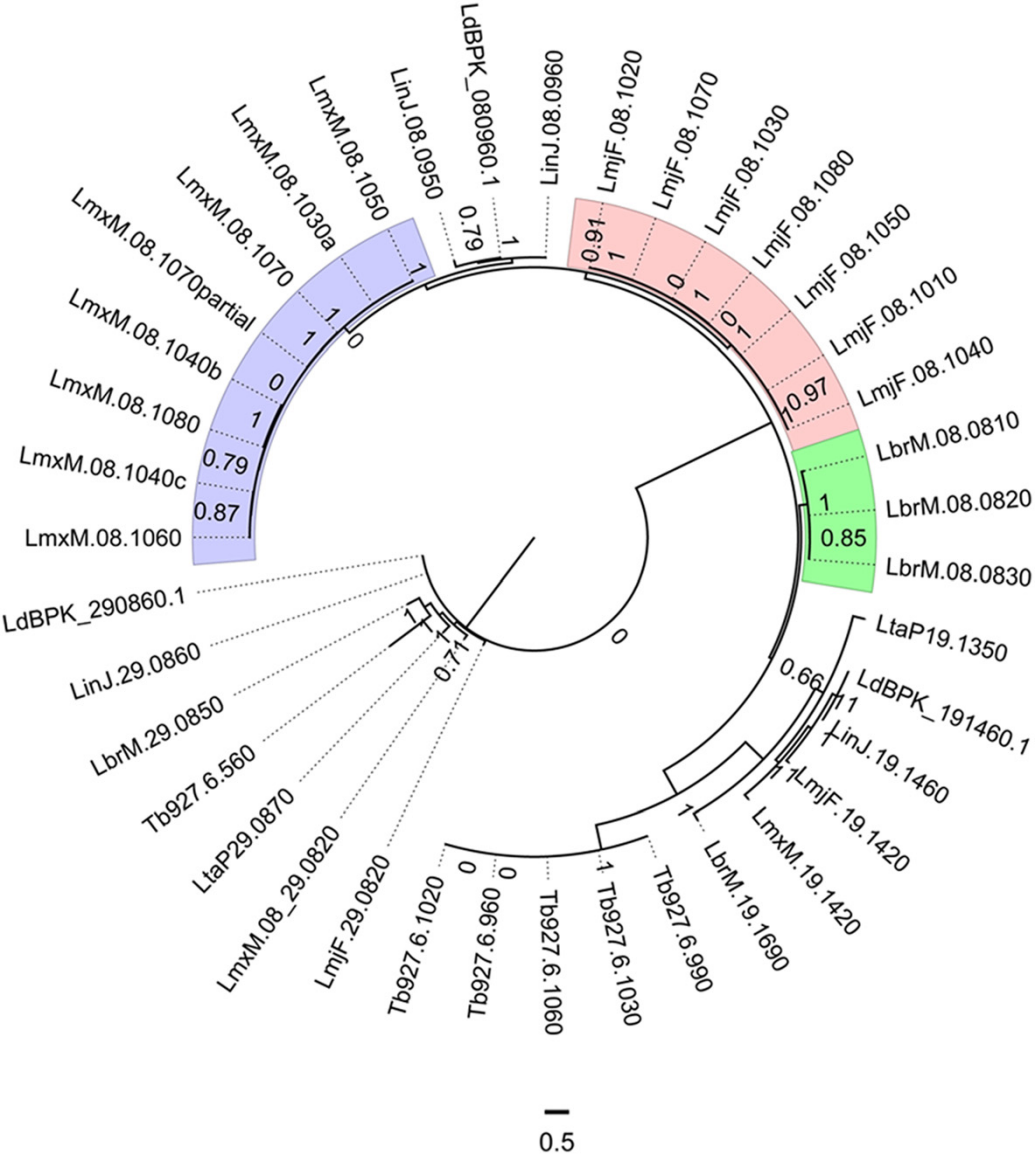
Cysteine peptidases phylogenetic relationship. Phylogenetic relationships for cysteine peptidases using *L. (Leishmania) major* seed protein LmjF.08.1070 under the WAG model. Numbers indicate support values computed by the approximate likelihood ratio test (aLTR). *Blue*, *rose* and *green* colored regions represent *L. (Leishmania) mexicana, L. (Leishmania) major* and *L. (Viannia) braziliensis* specific cathpesin-L expansions, respectively. Gene codes indicate the following species: LinJ: *L. (Leishmania) infantum*; LmxM: *L. (Leishmania) mexicana*; LmjF: *L. (Leishmania) major*; LdBPK: *L. (Leishmania) donovani;* LbrM: *L. (Viannia) braziliensis*; Lta: *L. (Sauroleishmania) tarentolae*

Our results also revealed species-specific expansions in cysteine peptidases (CPs) in *L. (Leishmania) mexicana, L. (Leishmania) major* and *L. (Viannia) braziliensis.* These expansions are located in tandem arrays in chromosome 8 (Fig. 7). Previous studies on Cathepsin-B have shown immunomodulatory roles suppressing the Th1 response, ensuring parasite survival in *L. (Leishmania) mexicana* and *L. (Leishmania) major* and that their activity could result in different disease phenotypes in both species [61, 62]. The corresponding phylogeny of cysteine peptidases showed that cathepsin-L genes are exclusively located in chromosome 8, cysteine peptidases A in chromosome 19, and cathepsin-B in chromosome 29.

*L. (Leishmania) mexicana, L. (Leishmania) major* and *L. (Viannia) braziliensis* present eight, seven and three expansions of Cathepsin-L, respectively (Fig. 7). These expansions are organized into gene arrays and share more than 70 % similarity at the protein level.

RNA-expression data for *L. (Leishmania) major* retrieved from the Trytrip database [17] shows that these Cathepsin-L genes have, on average, a 1.7-fold increase in amastigotes versus procyclic promastigotes and up to a 1.8-fold increase between metacyclic versus procyclic promastigotes, which suggests that Cathepsin-L expression is modulated during parasite development with expression increasing towards the infective and intracellular stages.

### Orthology relationships in *Leishmania*

Using BioPerl:Trees, we extracted orthologs and paralogs for each seed protein to analyze the ones that are unique in each species and to look at their respective homologs.

A total of 28 trees summarizing the relationships of 72 genes were species-unique (Additional file 1: Table S3). From these, 25 trees belonged to *L. (Viannia) braziliensis* and comprised TATE DNA transposons, SLACS, a phosphatidic acid phosphatase, and hypothetical proteins. The remaining trees belonged to a folate biopterin transporter, an oligosaccharyl transferase in *L. (Leishmania) donovani*, and a hypothetical protein in *L (Leishmania) major*. The absence of a greater number of species-specific trees reflects the high conservation between *Leishmania* proteomes and underscores the importance of species-specific expansions. Another possibility is the variance in assembly completeness of *Leishmania* genomes that can limit an accurate assessment of orthology and paralogy relationships.

We found 299 trees comprising 1519 genes across five human pathogenic *Leishmania* species without orthologs in *L. (Sauroleishmania) tarentolae*. Protein families in these trees include histone 4, k39 kinesin, calpain-like cysteine peptidases, a2-rel and hypothetical proteins (Additional file 1: Table S4).

Calpain-like cysteine peptidases are predicted to encode large proteins with potential functions in signal transduction, cytoskeletal remodeling and membrane attachment during *Leishmania* differentiation [63, 64].

Previous studies have shown that disruption by gene targeting of a2-rel-related genes in *L. (Leishmania) donovani* generated mutants with reduced infectivity in mice and limited their proliferation in culture [65]; however, their specific function has not been elucidated yet.

We found a total of 11 trees that were shared by species of the *Leishmania donovani* complex without orthologs in *L. (Leishmania) major, L. (Viannia) braziliensis* nor *L. (Sauroleishmania) tarentolae* (Additional file 1: Table S5). Among these genes we found the presence of the A2 gene family that is the prototype of genes involved in visceralization [66] and hypothetical proteins that remain to be characterized.

*Leishmania* species that are associated with CL include *L. (Viannia) braziliensis, L. (Leishmania) mexicana*, *L. (Leishmania) major* and occasionally *L. (Leishmania) infantum* [2]. We found a total of 15 trees specific for all these species comprising of 72 proteins, most of which are annotated as hypothetical (Additional file 1: Table S6).

## Conclusions

Our results indicate that gene expansions are a common trait in *Leishmania* genomes and represent an important force in the evolution of these parasites. Major species-specific expansions in genes mediating host-parasite interactions reflect genome complexity and evolutionary processes that influence the wide spectrum of diseases that are caused by different *Leishmania* species.

An important limitation of the current study is the different assembly completeness across the *Leishmania* genomes analyzed. It is known that repetitions and head-to-tail duplicated genes are likely to suffer from assembly and annotation errors leading to partial sequences that could have been excluded during the filtering steps. In this sense, it might be possible that the exact number of expanded genes may vary with subsequent improvements of the current genome assemblies.

The *Leishmania* metaphylome appears as a promising resource to aid the scientific community in understanding the complexity of host-parasite relationships and highlighting areas of interest for additional experimentation. Further studies are needed to determine the function of relevant hypothetical proteins that were identified here, characterize species-specific expansions, and employ transcriptomic data to complement our results.

## Additional file

Additional file 1:
**Supplementary tables.** (XLSX 148 kb)
